# Single-energy versus dual-energy imaging during CT-guided biopsy using dedicated metal artifact reduction algorithm in an in vivo pig model

**DOI:** 10.1371/journal.pone.0249921

**Published:** 2021-04-26

**Authors:** Thuy Duong Do, Julia Heim, Stephan Skornitzke, Claudius Melzig, Dominik F. Vollherbst, Michael Faerber, Philippe L. Pereira, Hans-Ulrich Kauczor, Christof Matthias Sommer

**Affiliations:** 1 Clinic for Diagnostic and Interventional Radiology, University Hospital Heidelberg, Heidelberg, Germany; 2 Department of Neuroradiology, University Hospital Heidelberg, Heidelberg, Germany; 3 Clinic for Radiology, Minimally-invasive Therapies and Nuclear Medicine, SLK Kliniken Heilbronn GmbH, Heilbronn, Germany; 4 Clinic for Diagnostic and Interventional Radiology, Klinikum Stuttgart, Stuttgart, Germany; Johns Hopkins School of Medicine, UNITED STATES

## Abstract

**Purpose:**

To evaluate dual-energy CT (DE) and dedicated metal artifact reduction algorithms (iMAR) during CT-guided biopsy in comparison to single-energy CT (SE).

**Methods:**

A trocar was placed in the liver of six pigs. CT acquisitions were performed with SE and dose equivalent DE at four dose levels(1.7–13.5mGy). Iterative reconstructions were performed with and without iMAR. ROIs were placed in four positions e.g. at the trocar tip(TROCAR) and liver parenchyma adjacent to the trocar tip(LIVER-1) by two independent observers for quantitative analysis using CT numbers, noise, SNR and CNR. Qualitative image analysis was performed regarding overall image quality and artifacts generated by iMAR.

**Results:**

There were no significant differences in CT numbers between DE and SE at TROCAR and LIVER-1 irrespective of iMAR. iMAR significantly reduced metal artifacts at LIVER-1 for all exposure settings for DE and SE(p = 0.02-0.04), but not at TROCAR. SNR, CNR and noise were comparable for DE and SE. SNR was best for high dose levels of 6.7/13.5mGy. Mean difference in the Blant-Altman analysis was -8.43 to 0.36. Cohen’s kappa for qualitative interreader-agreement was 0.901.

**Conclusions:**

iMAR independently reduced metal artifacts more effectively and efficiently than CT acquisition in DE at any dose setting and its application is feasible during CT-guided liver biopsy.

## Introduction

CT-guided biopsy enables accurate tumor localization, intraprocedural monitoring and confirmation of needle position directly before obtainment of histological samples with a reported accuracy of 86% - 98% [1–3]. CT scans with contrast agent did not achieve better biopsy results compared to non-contrast CT [1]. Contrast agent may only temporarily enhance the depiction of the lesion with the risk of disguising the subject lesion in the late phase. The visibility of liver lesions during CT-guided biopsy is significantly reduced by metal artifacts caused by the biopsy needle, leading to insufficient visualization especially for small lesions with diameters less than 3 cm or when using an intercostal or subcostal access [1]. As shown by Stattaus et al., the insufficient visualization of small lesions leads to an increased rate of false-negative biopsy results of 12.8% in comparison to lesions with good visibility. The visibility of liver lesions decreased significantly when approaching the lesion with the biopsy needle up to the critical moment when the needle reaches the lesion of interest [1]. While changing window settings can reduce metal artifacts induced by the biopsy needle, at the same time the soft tissue contrast and thus the visibility of the liver lesion is reduced. Alternatively, removing the canulae inside the trocar and using a smaller-gauge guide can reduce needle artifacts significantly [4].

In general, iterative reconstructions and dual-energy CT have shown to improve low-contrast detectability of liver lesions and studies show that it is possible to reduce tube current to half while maintaining equal low-contrast detectability when using iterative reconstruction [5, 6]. Furthermore, dedicated iterative Metal Artifact Reduction (iMAR) iteratively combines the algorithms of Normalized Metal Artifact Reduction (NMAR) and Frequency Split Metal Artifact Reduction (FSMAR) [7, 8]. When using iMAR, the metal implant/needle is segmented based on a threshold and the discrepancies in the sonogram caused by the high attenuation of the metal are replaced by linear interpolation to reduce metal artifacts, which is repeated iteratively. The final image is then calculated by combining the high- and low-pass filtered versions of the corrected and the original image. Based on the recent increases in computational power, the complex implementation of iMAR reconstructions can be performed within seconds, so that iMAR can also be used during time-critical operations, like puncture procedures [7–9]. In general, metal artifact reduction algorithms have been successfully applied for different implants e.g., hip prostheses, dental hardware, head CT after intracranial aneurysm coiling and clipping, and spinal hardware [10–13].

The present study aims to evaluate the use of dual-energy (DE) with and without iMAR for metal artifact reduction during CT-guided needle biopsy at different exposure settings in comparison to single-energy (SE) acquisitions.

## Materials and methods

Approvals of the regional and institutional veterinary committees of the University of Heidelberg were obtained. The ethics committee of the University of Heidelberg also approved this study. The regional executive board for agriculture and veterinary of Karlsruhe approved the experiments.

### Animal preparation

Six German landrace pigs with a body weight of 31–40 kg served as objects of investigation. Anesthesia was achieved with intravenous midazolam (0.4 mg/kg; Dormicum^®^, Roche, Basel, Switzerland), azaperone (6 mg/kg; Stresnil^®^, Janssen Animal Health, Beerse, Belgium) and ketamine (8–10 mg/kg; Ketanest-S^®^, Medistar, Hannover, Germany). All animals were intubated for breath control and a central venous catheter was positioned into the superior vena cava for medication access. For a secure muscle relaxation and optimal respiratory control, vecuronium (0.02 mg/kg; Vecuronium Inresa^®^, Inresa Arzneimittel GmbH, Freiburg, Germany) was administered. Constant narcose was achieved with sevoflurane (Sevoflurane, Baxter, Unterschleißheim, Germany) and oxygene-gas-mixture. Animals were in placed in supine position during the CT image acquisition.

A commercially available biopsy system (Spirotom^®^, Bioncise, Hasselt, Belgium) was placed into the right liver lobe under CT navigation and holding end-expiratory breath at 6 cm depth from cutis. The biopsy system consisted of a biopsy needle (22 cm length and 14-gauge shaft), a trocar (14.5 cm length and 13-gauge shaft) and a canulae (17 cm length and 14-gauge shaft). During imaging of the biopsy procedure the biopsy canulae was removed and the trocar tip position was verified. Only in the last step of retrieving the histological sample was the biopsy needle with helix tip used. At the tip, the needle contained a helix for a controlled cutting of the sample. The placement of the canulae (= stylet) was performed in a standardized manner with a depth of 6 cm from the skin surface in the liver parenchyma and parallel to the axial plane. CT scans were performed in three configurations: the trocar alone, trocar with canulae and the trocar with the biopsy needle with helix configuration.

### CT protocol and image reconstructions

Imaging was performed using a dual-source 64-MDCT scanner (Somatom Definiton Flash^®^, Siemens Healthineers, Erlangen, Germany). Each animal was scanned four times with a SE acquisition protocol as follows (tube potential/tube-current time product/volumetric CT dose index [CTDI_vol_]): 120 kV_p_/200 mAs/13.5 mGy, 120 kV_p_/100 mAs/6.7 mGy, 120 kV_p_/50 mAs/3.3 mGy, 120 kV_p_/25 mAs/1.7 mGy. Furthermore, four DE acquisitions were performed with the same CTDI_vol_ as for the SE acquisitions so that the applied dose would be comparable between the two. For DE scans, a tube voltage pair of Sn140/100 kV_p_ was selected with an additional tin filter of 0.1 mm for additional hardening of the 140 kV_p_ spectrum. Other parameters such as pitch (0.6), collimation (64x0.6 mm), gantry rotation time (0.28 s) and scan length (5 cm) were identical for all acquisitions and all animals. Scan direction was craniocaudal.

**Fig 1:**
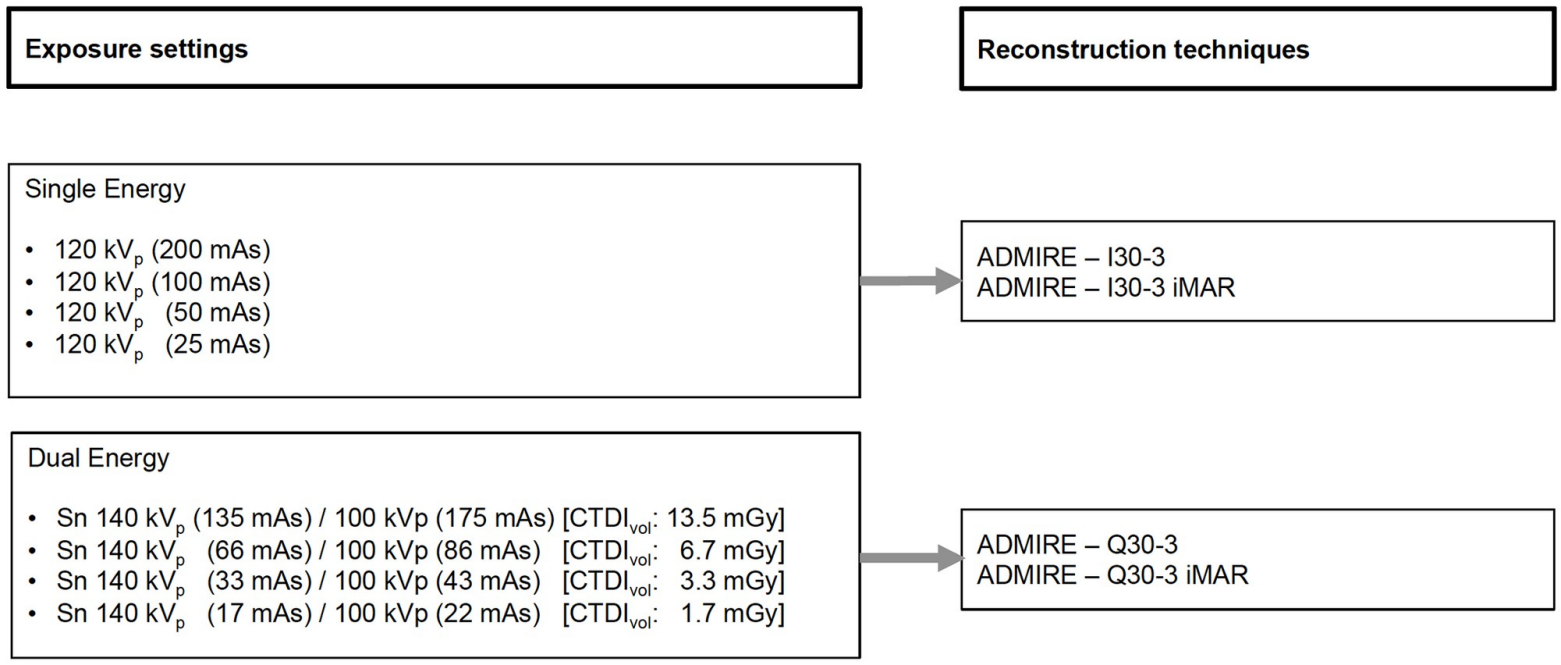
CT acquisition and reconstruction settings.

Iterative reconstructions (ADMIRE, Siemens Healthineers, Erlangen, Germany) were performed using a soft kernel for SE (I30-3) and DE (Q30-3) with a medium level of 3. For the DE acquisitions, linearly blended images at M0.5 was reconstructed. Each iterative reconstruction at any tube voltage/tube current combination was performed with and without dedicated iterative metal artifact reduction algorithm (iMAR, Siemens Healthineers, Forchheim, Germany) (Fig 1). Each iMAR reconstruction required less than a minute to obtain the corrected images. Slice thickness was 2 mm and increment was 1 mm.

Overall, 16 reconstructed datasets were analyzed for the comparison of SE CT versus linearly blended DE CT.

### Image analysis

Regarding needle configuration, solely the trocar without canulae was chosen for image analysis to match clinical practice, because during puncture procedures on patients the CT image acquisitions are performed without the biopsy needle in order to minimize metal artifacts.

Two independent observers conducted the quantitative evaluations. Qualitative analysis was performed twice by two observers, one with 5 and the other with 6 years of experience in abdominal and interventional radiology (Read 1 and Read 2).

#### Quantitative image analysis

**Fig 2:**
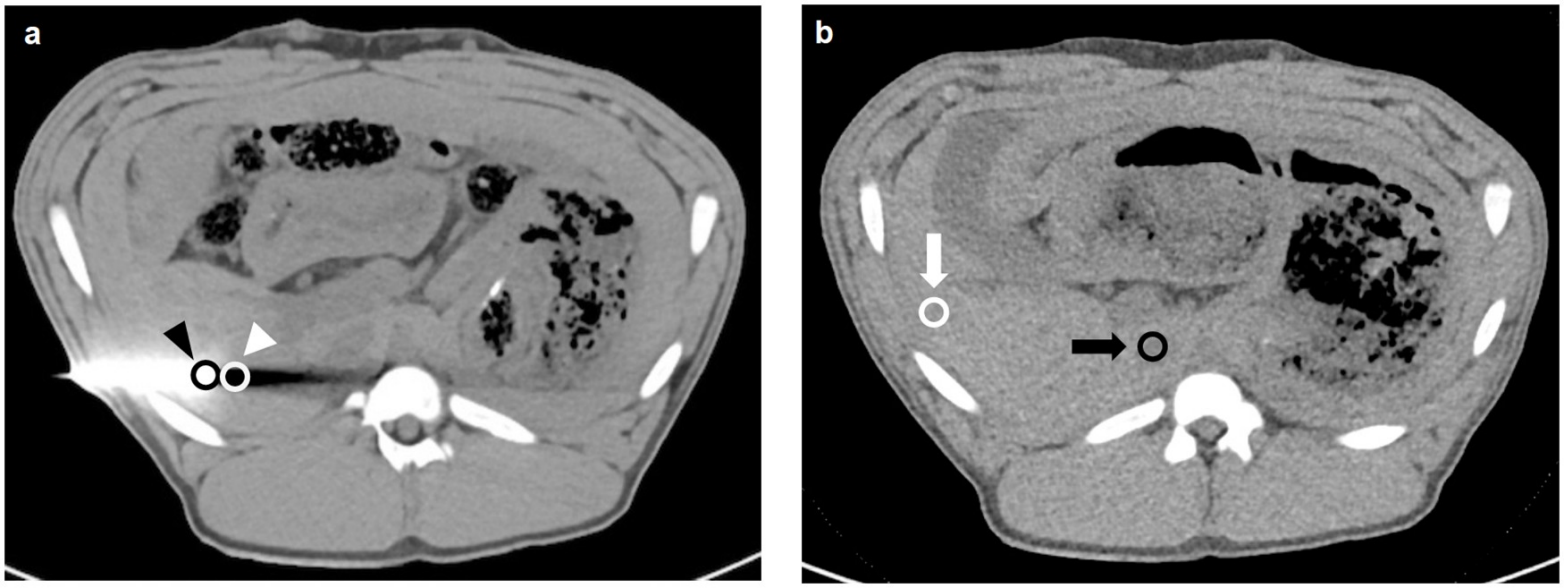
Image example for the placement of regions of interest. Axial image with trocar (a) with ROIs at the trocar tip (black arrowhead) and in the liver parenchyma adjacent to the trocar tip (white arrowhead). Axial slice without any visible artifacts (b) with ROIs in the liver parenchyma (white arrow) and inferior vena cava (black arrow).

To objectively evaluate the image quality and metal artifacts, images were transferred to a multi-modality workstation (syngo.via, Siemens Healthineers, Erlangen, Germany) and the investigation was performed using four regions of interest (ROIs). All ROI areas were 0.5 cm^2^ ± 0.04 cm^2^. The ROIs from each animal was intra-individually copied to all other reconstructions of the same animal. Means and standard deviations were calculated from the ROI measurements for density evaluation in Hounsfield Units (HU). ROIs were manually positioned at the tip of the trocar (TROCAR) and in the liver parenchyma bordering the tip of the trocar (LIVER-1) in extension of the puncture direction (Fig 2A) in the slice with the maximum visible extent of beam hardening, photon starvation, and other metal artifacts. Additionally, in a slice without the trocar and without any visible artifacts, ROIs were placed in the inferior vena cava. Further ROIs on the peripheral liver parenchyma (LIVER-2) were used for the evaluation of image noise and signal-to-noise ratio (SNR) (Fig 2B).

Image noise was defined as the standard deviation of the ROI LIVER-2. SNR and CNR were calculated as follows:

$$SNR=\frac{MEAN\left(LIVER-2\right)}{SD(LIVER-2)}$$ (1)

$$CNR=\frac{\left|MEAN\left(LIVER-1\right)-MEAN\left(LIVER-2\right)\right|}{SD(LIVER-2)}$$ (2)

#### Qualitative image analysis

The qualitative analysis was performed on a standard diagnostic workstation (Picture Archive and Communication System [PACS] Centricity, GE, Boston, USA). All reconstructions had a preset window center of 40 HU and window width of 400 HU. The observers were blinded concerning image acquisition (SE, DE, tube current) and reconstruction algorithm (with/without iMAR). During image evaluation the observers were permitted to scroll through the series and adapt window center and width. Criteria were overall image quality with a 5-point Likert scale (1-excellent, 2-good, 3-fair, 4-poor, 5-non-diagnostic) and artifact degree in the liver parenchyma bordering to the trocar tip (1-none, 2-mild, 3-moderate, 4-severe, 5-non-diagnostic). For the evaluation of new artifacts generated by iMAR, observers were shown iMAR and non-iMAR images and these were compared side-by-side (1-none, 2-peripheral or around the trocar, 3-peripheral and around the trocar, 4-severe and 5-non-diagnostic).

As a proof-of-principle the iMAR algorithm was applied to SE acquisitions during CT-guided biopsy (liver, pelvis, abscesses) in 12 patients and reconstruction time was measured for axial images with iMAR from the start of a new reconstruction to the completion of the series recontruction.

### Statistical analysis

Statistical analysis was calculated using Prism (Prism, 7.0b 2016, GraphPad Software, La Jolla, USA). The p-value was used descriptively and p<0.05 was considered as significant. A 95%-confidence interval was assumed. A non-normal distribution of the data was expected.

A non-parametric ANOVA Friedman test was used to compare different reconstruction algorithms of the same dose level and different dose levels of the same reconstruction type. Dunn’s test was used as a post hoc test for the analysis and correction of the multiple comparisons between each reconstruction algorithm and between dose level comparisons. These tests were applied in order to compare density measurements, SNR and noise. Quantitative interreader agreement was rated using the Bland-Altman analysis [14, 15].

Results of the qualitative analysis of overall image quality, artifacts bordering to the trocar tip and new artifacts by iMAR were grouped into dose levels and reconstruction algorithms. Dose levels and reconstruction algorithms were analyzed using the non-parametric ANOVA Friedman test. Dunn’s test was used as a post hoc test for multiple comparison correction. Qualitative interreader agreement was rated using Cohen’s kappa [16, 17].

## Results

### Quantitative image analysis

**Fig 3:**
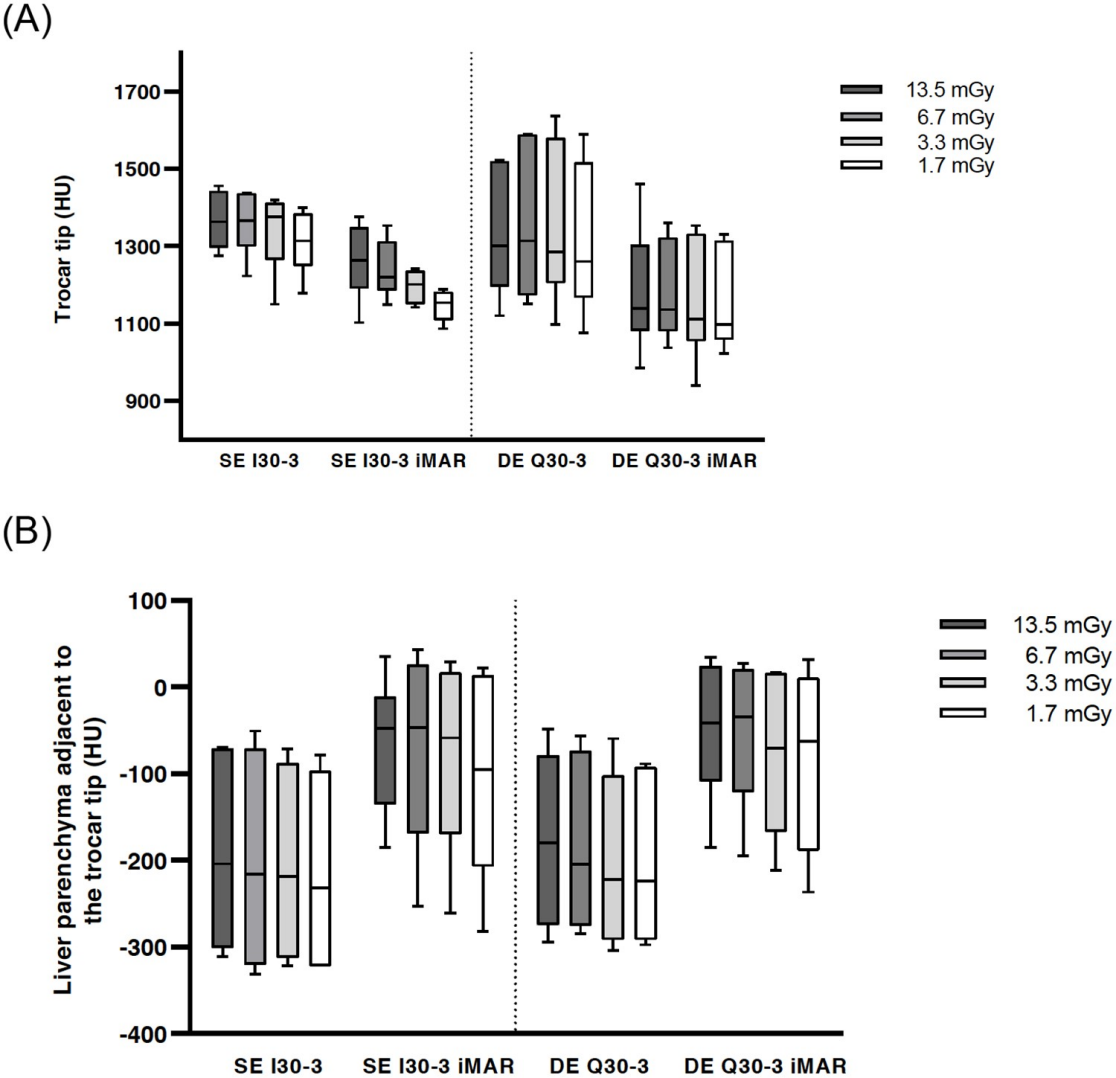
Boxplot of HU values at the trocar tip. No significance between DE vs. SE: I30-3 vs. Q30-3 and I30-3 iMAR vs. Q30-3 iMAR (a). Boxplot of HU values of liver parenchyma adjacent to the trocar tip showing significant metal artifact reduction with iMAR for SE and DE (b).

**Fig 4:**
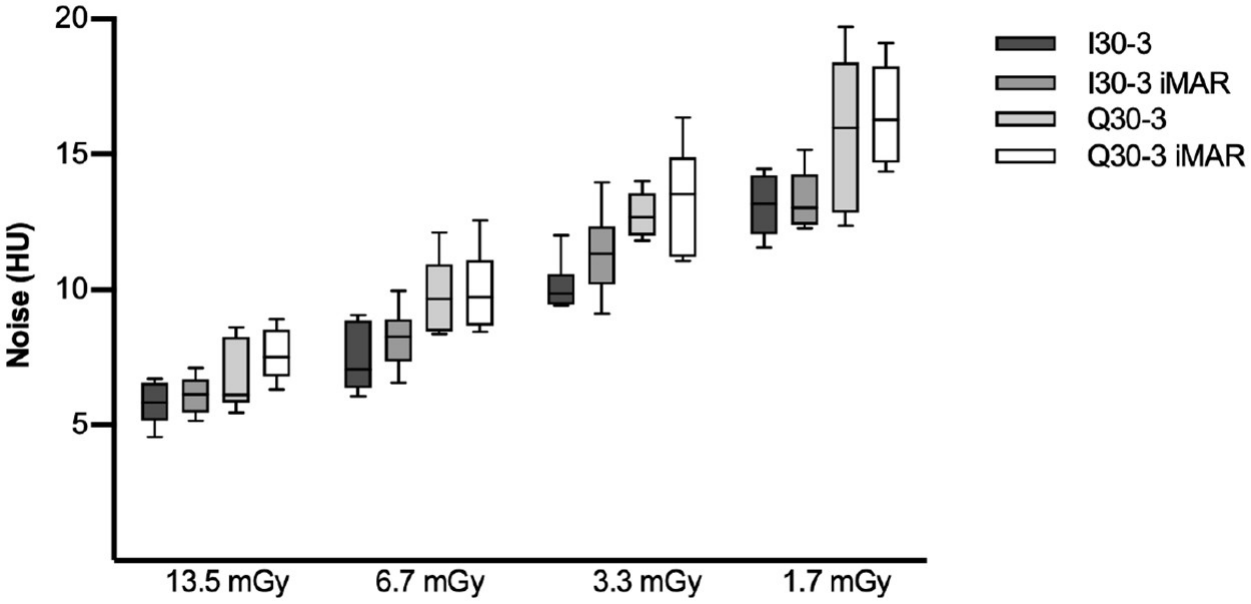
Boxplot of noise in an axial slice without artifacts. Noise is inversely proportional to dose level. Noise was lower, though not significantly so, for SE in comparison to DE.

Results of quantitative analysis of the trocar tip (TROCAR) and LIVER-1 are presented in Tables 1 and 2 and Figs 3 and 4.

**Table 1:** HU values at the trocar tip (TROCAR) with median and 1st and 3rd percentiles in brackets.

CTDI_vol_	SE I30-3	SE I30-3 iMAR	DE Q30-3	DE Q30-3 iMAR	p-value
**13.5 mGy**	1363 (1306;1431)	1264 (1228;1324)	1301 (1241;1465)	1139 (1116;1227)	0.0558
**6.7 mGy**	1366 (1331;1422)	1219 (1199;1284)	1314 (1208;1526)	1137 (1095;1276)	**0.0033**^1^
**3.3 mGy**	1377 (1315;1408)	1201 (1158;1234)	1285 (1246;1497)	1111 (1094;1274)	**0.0057**^2^
**1.7 mGy**	1314 (1281;1365)	1153 (1125;1174)	1261 (1208;1439)	1097 (1075;1259)	**0.0096**^3^
**p-value**	0.0558	**0.0321**^4^	0.3165	0.6679	

No significant effect for iMAR was found. In general no significant difference was found when comparing exposure settings except for SE I30-3 iMAR.

Dunn’s test corrected p-value for multiple comparisons:

^1^ 6.7 mGy

I30-3 vs. Q30-3 iMAR p-value: 0.0105.

^2^ 3.3 mGy

I30-3 vs. Q30-3 iMAR p-value: 0.0219.

^3^ No significance.

^4^ I30-3 iMAR

13.5 mGy vs. 1.7 mGy p-value: 0.0437.

**Table 2:** HU values at the liver parenchyma adjacent to the trocar tip (LIVER-1) with median and 1st and 3rd percentiles in brackets.

CTDI_vol_	SE I30-3	SE I30-3 iMAR	DE Q30-3	DE Q30-3 iMAR	p-value
**13.5 mGy**	-204 (-296;-82)	-48 (-106; -27)	-180 (-256;-101)	-41 (-80;13)	**0.0002**^1^
**6.7 mGy**	-216 (-313;91)	-47 (-130;17)	-204 (-264;-102)	-34 (-90;15)	**0.0002**^2^
**3.3 mGy**	-218 (-309;-102)	-58 (-133;9)	-222 (-279;-134)	-70 (-143;6)	**0.0002**^3^
**1.7 mGy**	-232 (-320;-115)	-95 (-179; 3)	-224 (-281;-118)	-62 (-158;-1)	**<0.0001**^4^
**p-value**	**0,0130**^5^	**0,0412**^6^	**0,0015**^7^	**0,0015**^8^	

iMAR increased reduced HU values for SE and DE. Acquisition mode (SE vs. DE i.e., SE I30-3 vs. DE Q 30–3 and SE I30-3 iMAR vs. Q 30–3 iMAR) did not influence HU values significantly.

Dunn’s test corrected p-value for multiple comparisons:

^1^ 13.5 mGy

I30-3 vs. I30-3 iMAR p-value: 0.0437.

I30-3 vs. Q30-3 iMAR p-value: 0.0437.

Q30-3 vs. I30-3 iMAR p-value: 0.0437.

Q30-3 vs. Q30-3 iMAR p-value: 0.0437.

^2^ 3.3 mGy

I30-3 vs. I30-3 iMAR p-value: 0.0219.

I30-3 vs. Q30-3 iMAR p-value: 0.0219.

^3^ 6.7 mGy

I30-3 vs. I30-3 iMAR p-value: 0.0219.

I30-3 vs. Q30-3 iMAR p-value: 0.0219.

^4^ 1.7 mGy

I30-3 vs. Q30-3 iMAR p-value: 0.0048.

Q30-3 vs. Q30-3 iMAR p-value: 0.0219.

^5^ I30-3

13.5 mGy vs. 1.7 mGy p-value: 0.0105.

^6^ I30-3 iMAR

6.7 mGy vs. 1.7 mGy p-value: 0.0437.

^7^ Q30-3

13.5 mGy vs. 1.7 mGy p-value: 0.0219.

6.7 mGy vs.1.7 mGy p-value: 0.0437.

^8^ Q30-3 iMAR

13.5 mGy vs. 3.3 mGy p-value: 0.0219.

13.5 mGy vs. 1.7 mGy p-value: 0.0437.

CT numbers at the trocar tip did not show any significant differences between the different reconstruction settings (SE I30-3, SE I30-3 iMAR, DE Q30-3, DE Q30-3 iMAR) for the highest dose CTDI_vol_ 13.5 mGy level, but iMAR images showed lower median values (SE I30-3 iMAR: 1264 HU and DE Q30-3 iMAR: 1139 HU) in comparison to non-iMAR images (SE I30-3: 1363 HU and DEQ30-3: 1301 HU). Significant differences in CT numbers were found regarding iMAR effects on the reconstructions of all other exposure settings with lower median values for iMAR images (Table 1 **right p-value column**). No significant differences in CT numbers were noted for the same reconstruction algorithm at different exposure settings except for SE I30-3 iMAR (Table 1 **p-values lowest row;** Fig 3A).

For LIVER-1 there was a significant difference in CT numbers for iMAR images (e.g., CTDI_vol_ 13.5 mGy: SE I30-3 iMAR [-204 HU] and DE Q30-3 iMAR [-180 HU] compared to non-iMAR images (SE I30-3 [-48 HU] and DE Q30-3 [-41 HU]) (Table 2 **right p-value column**). No significant differences were found concerning the acquisition mode DE vs. SE i.e., SE I30-3 vs. DE Q30-3 and SE I30-3 iMAR vs. DE Q30-3 iMAR. For each acquisition mode, significant differences were found between the highest dose levels at 13.5/6.7 mGy vs. the lowest dose level 1.7 mGy. (p = 0.0015–0.04) (Table 2 **p-value lowest row**).

In the axial slide without any artifacts, HU values of the inferior vena cava showed no significant differences in regard to the acquisition mode (DE vs. SE) and exposure settings. The maximum HU difference measured for the inferior vena cava was 3 HU. For liver parenchyma, the HU values for SE images were lower in comparison to DE images. However, statistical tests showed significant differences between iMAR and non-iMAR images and the maximum difference of HU values was 4 HU. No differences were noted between different exposure settings for the liver parenchyma in the slice without any visible artifacts.

High exposure settings showed significant lower noise compared to low dose levels. Higher noise was observed for DE in comparison to SE, though not at a statistical significance level i.e. I30-3 vs. Q30-3 and I30-3 iMAR vs. Q 30–3 iMAR. No difference was found between iMAR vs. non-iMAR images, although in combination with SE and iMAR a significant difference was found between SE I30-3 and DE Q30-3 iMAR (Fig 4).

**Fig 5:**
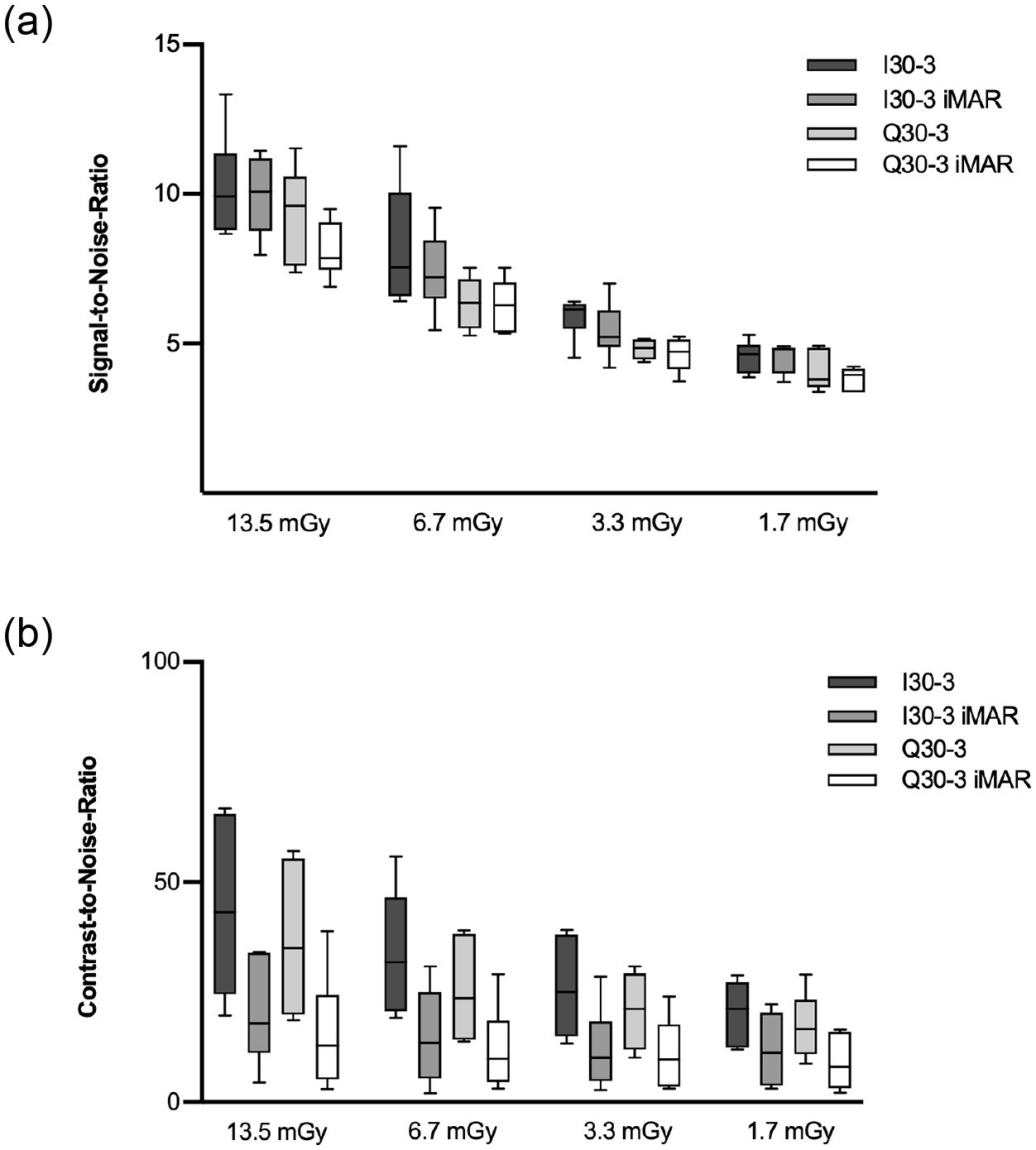
Boxplot of SNR (a) and CNR (b). SNRs were best for high dose levels and in general higher for SE in comparison to DE, but there was no significant difference between SE I30-3 vs. DE Q30-3 and SE I30-3 iMAR vs. DE Q30-3 iMAR. CNR was comparable for DE vs. SE and iMAR vs. non-iMAR, though with slightly lower CNR for iMAR without statistical significance.

With decreasing dose exposure settings the SNR decreased for all reconstruction algorithms, but the decrease was only statistically significant for the comparison of 13.5 mGy vs 1.7 mGy (p<0.008). SNR for SE (3.72–13.33) was higher than DE (3.37–11.53) irrespective of dose level and iMAR, but both comparisons were not statistically significant (Fig 5a).

CNR correlated positively with dose exposure e.g. CNR_13.5mGy_:44.02 vs. CNR_1.7mGy_:9.03 (p = 0.039). CNR was lower for DE (9.03–36.86) than SE (11.98–44.02) acquisitions at different dose levels, but the difference was not statistically significant. For all dose settings iMAR showed lower CNR than non-iMAR images without statistical significance (Fig 5b).

### Qualitative image analysis

**Fig 6:**
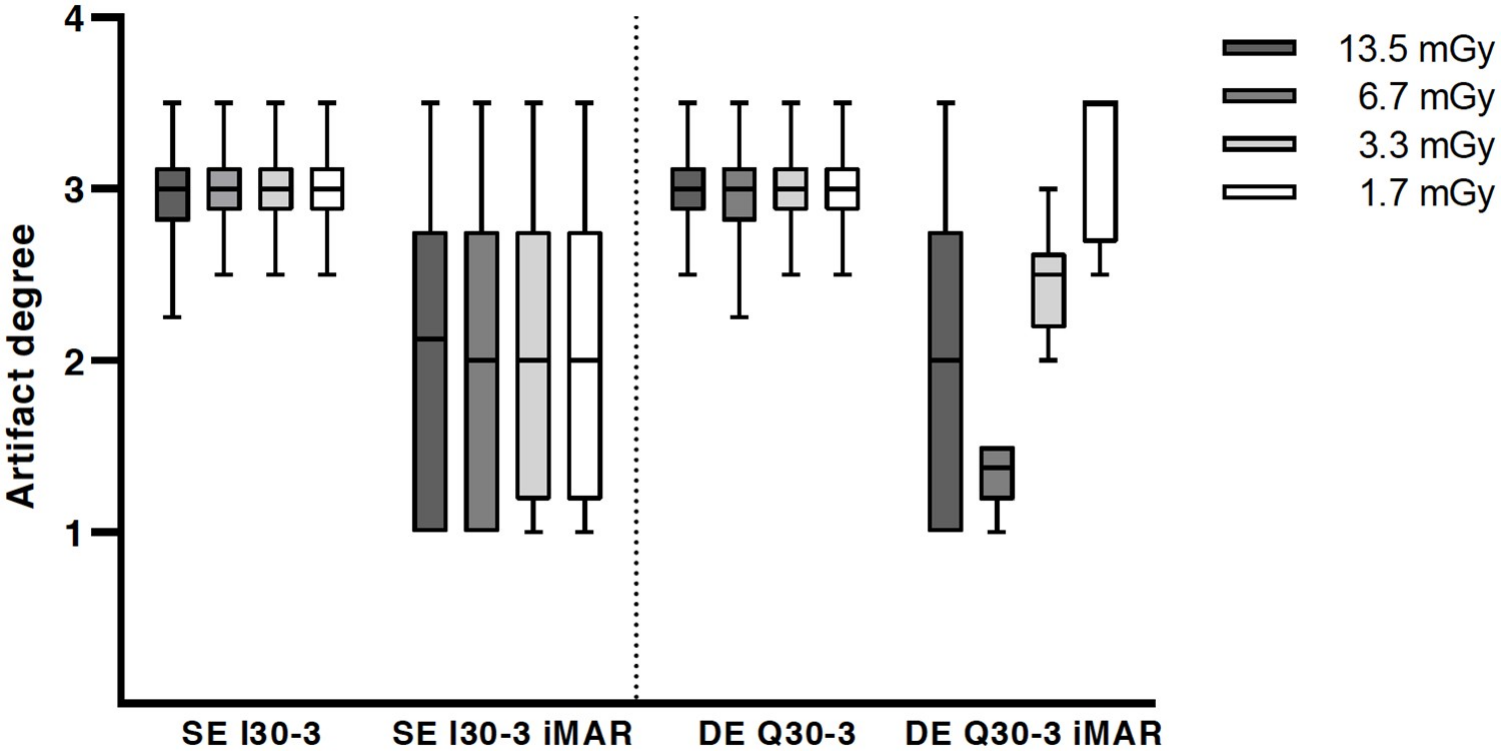
Qualitative analysis of artifact degree with significant metal artifact reduction for iMAR.

Results of the qualitative analysis are shown in Fig 6.

**Fig 7:**
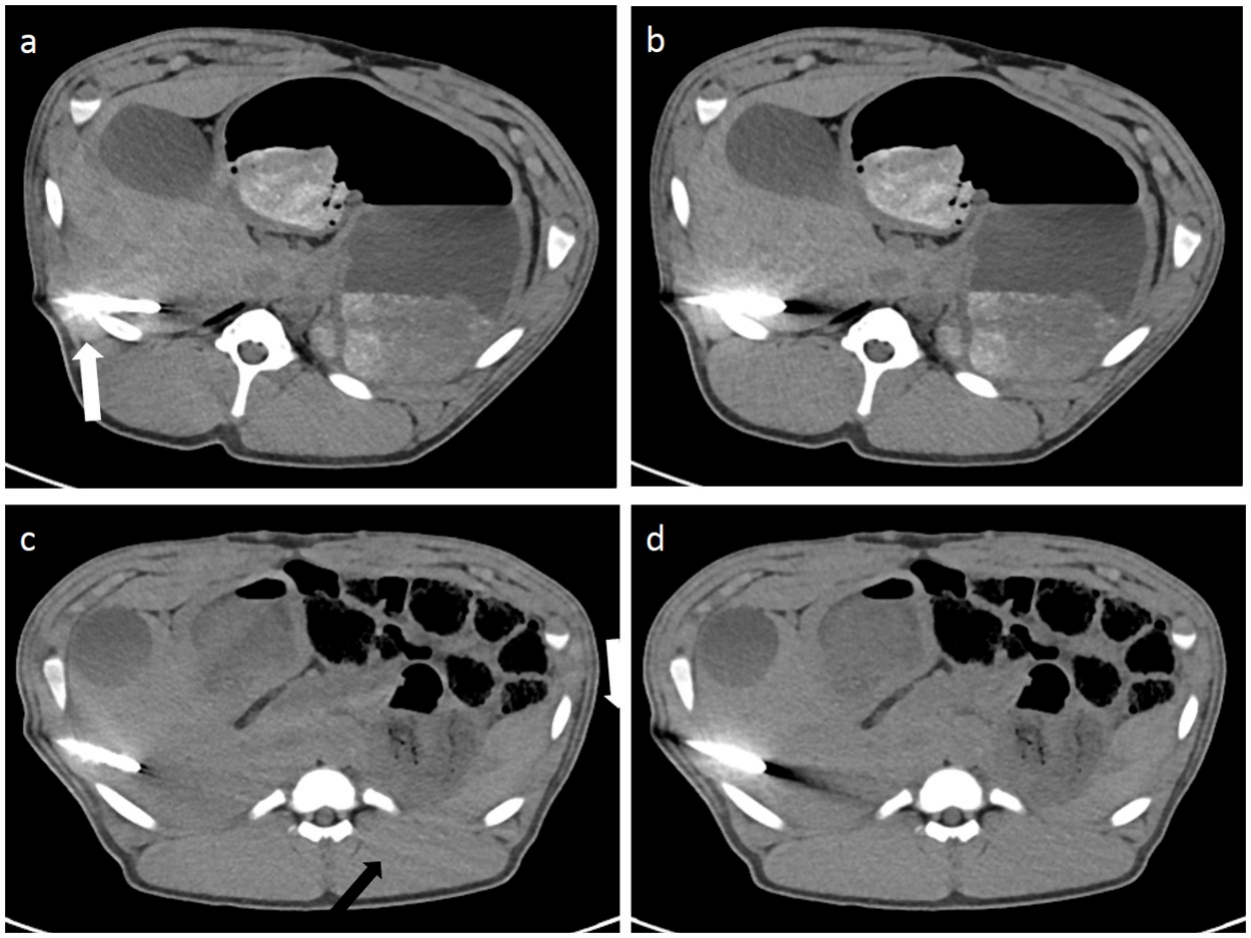
Image examples of iMAR effects and of newly generated artifacts by iMAR. iMAR images showed significantly less artifacts around the trocar tip and in the liver parenchyma bordering to the trocar tip (a and c). Blooming artifacts (white arrow) were most prominent around the trocar (a) in comparison to non-iMAR images (b). Peripheral streaks (black arrows) were observed at the image periphery with iMAR (c) in comparison to the corresponding non-iMAR image (d).

Overall image quality was considered to be significantly superior for higher dose levels (CTDI_vol_ 13.5 mGy and 6.7 mGy) in comparison to lower exposure settings (3.3 mGy and 1.7 mGy) for all reconstruction algorithms and acquisition modes (p<0.0001). There were no improvements of overall image quality for DE in comparison to SE irrespective of iMAR. However, a significant improvement of artifact degree in the liver parenchyma adjacent to the trocar tip was noted for iMAR images vs. non-iMAR images in the Friedman test, but statistically there was no improvement in the post hoc test (Fig 6). The dose level did not significantly influence the artifact degree in the liver parenchyma. For all iMAR images new artifacts were noted, with mostly blooming artifacts occurring around the trocar. Dark streak artifacts in the image periphery were also noted, but less common. DE acquisition did not influence the extent of the newly generated artifacts (Fig 7). The peripheral artifacts were fewer in the lower exposure settings.

#### Translation into clinical setting

**Fig 8:**
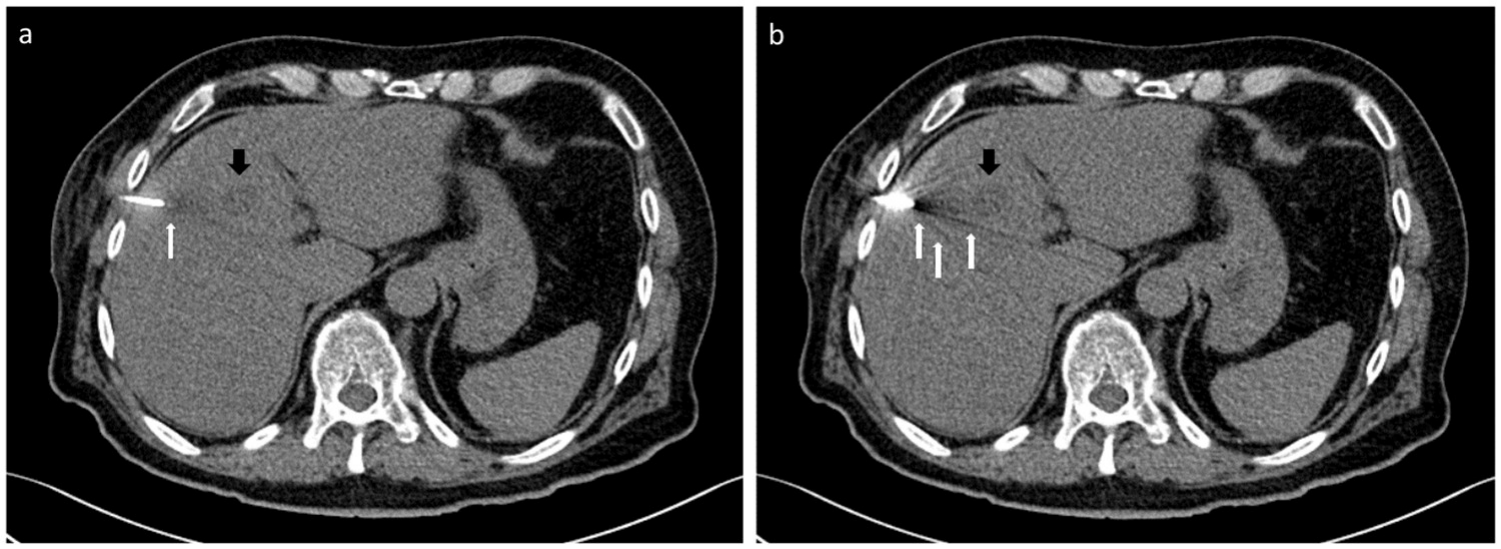
Images of a patient during CT-guided biopsy of a liver metastasis of a chordoma (black arrow) in SE technique displaying less streak artifacts at the crucial part of liver parenchyma bordering to the trocar (white arrow) on images with iMAR (a) than without iMAR (b).

The proof-of-principal clinical application of the iMAR algorithm in CT-guided intervention in 12 patients showed a mean reconstruction time of 12.2 ± 4.0 seconds for an average number of 41.1 ± 30.7 images of a CT helical scan in puncture mode. Proof-of-principle images with iMAR (Fig 8a) displayed less streak artifacts at the LIVER-1 position, a slightly better delineation of the trocar tip itself and improved visibility of the liver lesion in comparison to conventional images without iMAR (Fig 8b). In all cases a histological or microbiological sample could successfully be obtained.

#### Interreader agreement

The mean bias in the Blant-Altman analysis for LIVER-1 was from -8.43 to 0.12 HU (depending on acquisition mode) and 95% limits of agreement ranged from -29.19 to 17.52 HU.

Cohen’s kappa for qualitative analysis was 0.901 and according to the Landis and Koch classification it approached the classification of perfect.

## Discussion

There is a broad range of methods available to improve liver biopsies, i.e., guidance with ultrasound/computed tomography/MRI, cutting needles, automatic devices [18–20]. In the present study, DE CT linearly blended images alone were not superior in reducing metal artifacts sufficiently nor in improving image quality in comparison to SE CT. However, iMAR effectively and efficiently reduced streak artifacts in LIVER-1, the most critical part of the liver parenchyma ahead of the trocar tip during CT-guided biopsy.

The image acquisition in DE technique alone does not improve image quality (i.e. SNR or CNR) which is accordance with previous studies [21, 22]. For DE CT with rapid voltage switching a higher radiation exposure has to assumed [23], while in this study the tube currents of the dual-source DE CT could be matched to achieve a similar dose to the SE acquisition. Schenzle et al. demonstrated an improved contrast-to-noise-ratio at equivalent dose in an Alderson phantom for thoracic imaging [24]. However, the results of this in vivo study showed that for all exposure settings, the SNRs and CNRs of DE images were lower than those of SE images with equalized CTDI_vol_. Consequently, DE acquisition alone did not sufficiently improve image quality nor did it reduce metal artifacts. However, the use of dual-energy imaging offers the possibility of additional image post-processing such as the calculation of virtual monochromatic imaging (VMI). Many studies have been performed to analyze the ability of VMI to reduce metal artifacts, mostly in combination with dedicated iterative metal artifacts (iMAR, OMAR, MAR depending on vendor’s labeling), but without an explicit comparison of the effect of metal artifact reduction based on VMI vs. effects yielded by dedicated metal artifact reduction algorithms separately. This has to be taken into account as dedicated metal artifact reduction algorithms are regularly not included in the standard post-processing equipment, but often require the additional purchase of a software package, which has economic implications. Those few studies investigating VMI effect and iMAR effect separately showed controversial results for the role of VMI for metal artifact reduction, but all stated that the dedicated metal artifact reduction algorithm was an effective tool and superior in its performance for the reduction of metal artifacts [25–28]. In general when using VMI, high keV extrapolations are recommended for metal artifact reduction but this results reduced soft tissue contrast and thus, cannot be transferred to CT guided liver biopsies without hesitation [29].

iMAR was an effective method to improve image quality in this study, especially regarding photon starvation artifacts at LIVER-1, which are affecting the visibility of the biopsy pathway during CT-guided biopsy. In general, the liver parenchyma adjacent to the trocar tip is the most important area of interest to determine further biopsy proceedings. However, the delineation of the trocar tip and reduction of the surrounding beam hardening artifacts along the trocar was not improved by iMAR. New artifacts were even observed for iMAR, with additional blooming artifacts around the trocar and occasional dark streaks in the image periphery. However, no new artifacts occurred in the liver parenchyma bordering to the trocar tip, thus no interference with puncture procedure would be expected. The occurrence of new artifacts might be explained by the choice of iMAR algorithm, which was originally developed for dorsally instrumented spondylodesis screws, and used here for approximation of a trocar. Also, newly generated artifacts at the image periphery were noted less often for iMAR images with lower exposure settings. The reason for that might have been that with lower exposure settings the image quality was generally reduced, making it more difficult to accurately spot these added artifacts.

In this study the canula was removed to reduce metal artifacts and only the trocar remained in the liver during imaging. However, removing the canula conveys the risk of air embolism and might not be suitable for chest punctures. Generally, an injection cap was used to prevent air suction with the canulae removed.

This study has some limitations. The study was not performed in humans but in an animal model to enable an intraindividual comparison of different acquisition modes and dose settings. Moreover, the multiple CT image acquisitions on human subjects would have resulted in an excessive radiation exposure. Furthermore, the puncture samples were not evaluated histologically to rate the probe quality as the animal liver did not contain any liver malignancies, which is one essential limitation of the study. The methodology of this study is generally more suited to assess the visibility of the liver parenchyma ahead of the trocar tip, and not the lesion itself, to ensure a safe puncture procedure with a clear pathway to the lesion when using anatomic landmarks for biopsy. The inferior vena cava was used as a substitute for a liver lesion in an attempt to mitigate this limitation. Further investigations should focus more closely on the delineation of actual liver malignancies in a clinical setting during biopsy procedures.

Cutting needles achieved adequate samples and higher specific diagnosis rates than fine needle aspiration, especially with bigger diameters [2, 3]. However, these biopsy needles also required appropriate trocars. The bigger the trocar, the more metal artifacts were observed. In this study a trocar of 13-gauge was used instead of a standard 18-gauge, thus in a regular setting fewer metal artifacts can be expected as well.

## Conclusion

In summary, DE CT linearly blended images alone did not show an advantage in image quality and metal artifact reduction compared to SE CT. iMAR was feasible for metal artifact reduction and improved image quality in the critical liver parenchyma during CT-guided biopsy independent of image acquisition. Therefore, SE CT in combination with iMAR is recommended during liver biopsies, if no additional DE CT post-processing is used.
